# Genome-Wide Association Analysis and Genomic Prediction of *Mycobacterium avium* Subspecies *paratuberculosis* Infection in US Jersey Cattle

**DOI:** 10.1371/journal.pone.0088380

**Published:** 2014-02-11

**Authors:** Yalda Zare, George E. Shook, Michael T. Collins, Brian W. Kirkpatrick

**Affiliations:** 1 College of Agricultural and Life Sciences, Department of Animal Sciences, University of Wisconsin-Madison, Madison, Wisconsin, United States of America; 2 College of Agricultural and Life Sciences, Department of Dairy Science, University of Wisconsin-Madison, Madison, Wisconsin, United States of America; 3 School of Veterinary Medicine, Department of Pathobiological Sciences, University of Wisconsin-Madison, Madison, Wisconsin, Unites States of America; Indian Institute of Science, India

## Abstract

Paratuberculosis (Johne’s disease), an enteric disorder in ruminants caused by *Mycobacterium avium* subspecies *paratuberculosis* (*MAP*), causes economic losses in excess of $200 million annually to the US dairy industry. To identify genomic regions underlying susceptibility to *MAP* infection in Jersey cattle, a case-control genome-wide association study (GWAS) was performed. Blood and fecal samples were collected from ∼5,000 mature cows in 30 commercial Jersey herds from across the US. Discovery data consisted of 450 cases and 439 controls genotyped with the Illumina BovineSNP50 BeadChip. Cases were animals with positive ELISA and fecal culture (FC) results. Controls were animals negative to both ELISA and FC tests that matched cases on birth date and herd. Validation data consisted of 180 animals including 90 cases (positive to FC) and 90 controls (negative to ELISA and FC), selected from discovery herds and genotyped by Illumina BovineLD BeadChip (∼7K SNPs). Two analytical approaches were used: single-marker GWAS using the GRAMMAR-GC method and Bayesian variable selection (Bayes C) using GenSel software. GRAMMAR-GC identified one SNP on BTA7 at 68 megabases (Mb) surpassing a significance threshold of 5×10^−5^. ARS-BFGL-NGS-11887 on BTA23 (27.7 Mb) accounted for the highest percentage of genetic variance (3.3%) in the Bayes C analysis. SNPs identified in common by GRAMMAR-GC and Bayes C in both discovery and combined data were mapped to BTA23 (27, 29 and 44 Mb), 3 (100, 101, 106 and 107 Mb) and 17 (57 Mb). Correspondence between results of GRAMMAR-GC and Bayes C was high (70–80% of most significant SNPs in common). These SNPs could potentially be associated with causal variants underlying susceptibility to *MAP* infection in Jersey cattle. Predictive performance of the model developed by Bayes C for prediction of infection status of animals in validation set was low (55% probability of correct ranking of paired case and control samples).

## Introduction

Paratuberculosis or Johne’s disease (JD) is a chronic bacterial infection of the gastrointestinal tract caused by *Mycobacterium avium* subspecies *paratuberculosis* (*MAP)*. *MAP* is contagious; infected animals expose their cohorts to the pathogen by shedding bacterium into their colostrum, milk or feces [1]. In cattle, young calves are at the highest risk for acquiring *MAP* infection [2]. The major route of *MAP* transmission is fecal-oral [3]. *MAP* is a slow-growing intracellular bacterium; infected animals remain asymptomatic for 2 to 10 years before showing clinical signs of the infection. Clinical signs of JD in *MAP-*infected dairy cattle usually appear after 2^nd^ or 3^rd^ lactation and include poor nutrient uptake, severe diarrhea, progressive weight loss, low milk production and eventually death [4]. There is currently no cure for this disease. The NAHMS Dairy 2007 study estimated the apparent herd-level prevalence of *MAP*-infected herds in the top 17 US dairy states to be at least 68% based on recovery of viable *MAP* in environmental fecal samples [5]. In a recent study, the true herd-level prevalence of *MAP* infection in these herds was estimated to be 91.1% [6]. JD is a common disease in countries with a significant dairy industry [7] and causes a negative impact on the global economy [8], [9].

JD, like most other complex diseases is multi-factorial i.e. under the influence of both genetic and environmental factors. Studies have shown that susceptibility to JD is heritable with the estimates ranging from 0.03 to 0.28 in cattle [10], [11], [12], [13], [14], [15], [16], [17].

Crohn’s disease (CD) is an inflammatory bowel disease (IBD) in humans with manifestations similar to those of JD in cattle. *MAP* has been found in some patients with CD [18], however a causal link between the two has not been demonstrated. In the past few years, genome-wide association studies (GWAS) have been applied widely to decipher the genetic basis of complex traits and diseases in human. Using this approach for IBD has resulted in identification of 163 loci conferring risk of CD and ulcerative colitis (another common form of IBD) [19].

In recent years, availability of the BovineSNP50 platform for genotyping ∼54,000 SNPs across the Bovine genome [20] has facilitated GWAS in cattle. Six GWAS seeking genomic regions underlying susceptibility or tolerance to infection with *MAP* in Holstein have been performed to date [21], [22], [23], [24], [25], [26]. Various loci on multiple chromosomes have been reported for association with susceptibly to *MAP* infection in Holsteins. Jersey is the second most common dairy breed after Holstein in the United States. This is the first GWAS for susceptibility to paratuberculosis infection in the Jersey breed. Our objectives were to identify genomic regions that underlie susceptibility to infection with *MAP* as well as development of a multi-marker model to be used in genomic selection against susceptibility to *MAP* infection in Jersey cattle.

## Materials and Methods

### Ethics statement

The University of Wisconsin-Madison College of Agricultural and Life Sciences Animal Care and Use Committee approved the procedures used with animals in this experiment.

### Resource population

Blood and fecal samples were obtained from ∼5,000 mature cows (minimum age of 20 months) from 30 commercial Jersey herds throughout the US in a retrospective cross-sectional design. Nomination of herds for this study was based on the prevalence of JD evidenced by the herd’s owner or veterinarian. Sampling was performed selectively for three herds and completely (whole herd) for the remaining 27 herds. Samples were shipped in insulated containers with cold packs by overnight courier to the Johne’s Testing Center of the School of Veterinary Medicine at the University of Wisconsin-Madison and processed upon receipt (separation of plasma, collection of buffy coats for DNA extraction). Serum was held at 4°C and fecal samples at –20°C until testing. All blood samples were tested within 7 days by a serum ELISA (JTC-ELISA) [27] with 30% sensitivity and > 99% specificity relative to fecal culture [28].

ELISA optical density (OD) values for each serum sample were converted to sample to positive ratios (S/P) using [OD_Sample_ – OD_Negative control_]/[OD_Positive control_ – OD_Negative control_] [29]. ELISA S/P ratios were categorized as negative (0 to 0.09), suspect (0.10 to 0.24), low positive (0.25 to 0.39), positive (0.40 to 0.99), and strong positive (≥1.00) as suggested [29] (Table 1). Animals categorized as low-positive, positive or strong-positive were all considered to be ELISA-positive. Within-herd apparent prevalence (number of test-positive animals divided by total animals in the herd) varied from 0.03 to 0.30 based on ELISA results. Within herds, for each ELISA-positive cow, two ELISA-negative cows (matched on birth date) were selected. All ELISA-positive and selected ELISA-negative cows were also tested for evidence of *MAP* infection by fecal culture (FC) [30] (Table 1). The sensitivity and specificity of fecal culture have been estimated to be approximately 74% and 100% for detection of infectious cows, respectively [31].

**Table 1:** Cross-tabulation of serum ELISA scores by fecal culture test results.

ELISA category^1^	Fecal No test	Fecal Negative	Fecal Suspect	Fecal Positive	Clinical^2^	Total
**No test**	-	1	-	2	-	3
**Negative** (0–0.09)	2,294	1,860	-	201	-	4,355
**Suspect** (0.10–0.24)	19	16	-	13	1	49
**Low positive** (0.25–0.39)	2	36	3	90	-	131
**Positive** (0.40–0.99)	-	30	2	139	1	172
**Strong positive** (≥1)	-	12	1	280	-	293
**Total**	2,315	1,955	6	725	2	5,003

1 Range of numbers in each category is sample/positive ratio.

2 Animals with clinical signs of Johne’s disease.

### Discovery data

In total, 1,000 cows including 500 cases and 500 controls were selected for discovery purpose (objective 1). Cases consisted of animals with positive results to both ELISA and FC (ELISA+/FC+). Controls were cows testing negative to both ELISA and fecal culture (ELISA-/FC-) and matched with cases on herd and birth date. In a previous study [32] using a temporal clustering approach, we showed that *MAP*-infected animals were significantly clustered by birth date within dairy herds. This finding strengthens the hypothesis of non-uniform exposure to *MAP* in dairy herds. The choice of matching cases and controls on their birth dates was to ensure similar exposure to the pathogen thus reducing the environmental noise. Considering lack of a gold standard for diagnosis of *MAP* infection, we used the results of ELISA and FC tests in combination for defining *MAP* infection status. The rationale was to increase certainty with which these phenotypes represent the true infection status of animals.

### Validation data

To validate the results of discovery GWAS, an additional 200 animals were selected from the original herds. All eligible ELISA+/FC+ animals and their matching ELISA-/FC- were already used in the discovery stage. The remaining test-positive animals were either ELISA+ or FC+. FC+ cows that were not used in the discovery stage were used as cases for validation (n = 100). This choice was made to reduce classification bias, as fecal culture is twice as sensitive as ELISA. Controls were ELISA-/FC- also matched with cases on herd and birth date (n = 100).

### Genotyping and quality control

Genomic DNA of discovery and validation animals was extracted from buffy coats using a variation of the typical proteolytic digestion and organic extraction method [33]. The purity (A260/A280) of DNA samples was assessed by spectrophotometry. DNA samples were also quantified using PicoGreen® dsDNA (Invitrogen) and adjusted to 50 ng/µl prior to genotyping.

Discovery cases (n = 500) and controls (n = 500) were genotyped for 54,609 single-nucleotide polymorphisms (SNPs) at the biotechnology center of the University of Wisconsin-Madison using the Bovine 50K SNP BeadChip (Illumina Inc, San Diego, CA) [20]. The genotypes were assessed for quality control (QC) criteria including animal call rate (> 90%), SNP call rate (> 95%), minor allele frequency (MAF) (> 0.01) and deviation from Hardy-Weinburg equilibrium (HWE) (P-value <1×10^−6^). A total of 22,022 SNPs failing one or more criteria were removed. Among the excluded SNPs 10,499 had MAF<0.01, 7,471 SNPs had call rate <95% and 4,052 SNPs deviated from HWE. The final data consisted of 889 animals (450 cases and 439 controls) and 32,587 SNPs.

For the validation data, 91 cases and 91 controls passed the DNA quality assessment. The BovineLD BeadChip (Illumina Inc, San Diego, CA) including 6,909 SNPs was used for genotyping [34]. A QC analysis was performed with the criteria of animal call rate (> 90%) and SNP call rate (> 95%). A total of 180 animals (90 cases, 90 control) and 6,796 SNPs passed QC. All QC procedures were done in R GenABEL package [35].

### Imputation

BEAGLE (v 3.3.2) was used to impute missing genotypes in the discovery and validation data [36]. The average missing rate per SNP in the discovery data after QC was ∼ 1%. Allelic R^2^ was estimated by BEAGLE based on genotype probabilities as an indicator of imputation accuracy. The average allelic R^2^ of 0.99 for imputed genotypes in the discovery data suggested high accuracy of imputations. Out of 6,796 SNPs in the validation set, 5,424 were common across LD and 50K chips. The genotypes of common SNPs were used to infer haplotypes and impute 27,163 SNPs unique to 50K panel for the validation data set. The average allelic R^2^ of 0.96 indicated high accuracy of imputed genotypes.

### Population stratification

Differences in allele frequencies between subpopulations of admixed populations can lead to false associations in GWAS [37]. To find genetic outliers, the genomic kinship was computed between all pairs of animals using the *ibs* function in the R GenABEL package [35]: 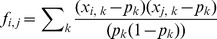
(1)where *f_i, j_* is the genomic kinship (identical-by-state) between animal *i* and *j*, *k* ranges from 1 to N (number of autosomal SNPs), *x_i,k_* or *x_j, k_* is the genotype of *i^th^* or *j^th^* animal for *k* ^th^ SNP (coded as 0, ½ and 1) and *p_k_* is the allele frequency at the *k^t^* ^h^ SNP. The kinship matrix was transformed to a distance matrix (0.5 – kinship) and principal components (PCs) of variation of the genomic distance matrix were calculated using the *cmdscale* function. The first two PCs (PC1 and PC2) were used to obtain the classical multi-dimensional scaling (MDS) plots. All population stratification procedures were performed within the R GenABEL package.

### Statistical analyses

**Single-marker GWAS.** Genome-wide association analysis was carried out based on regression of phenotypes (susceptibility to *MAP* infection) on the genotypes of animals for one SNP at a time. For single-marker GWAS, we used a three-step approach referred to as genomic GRAMMAR-GC (Genome-wide Rapid Association using Mixed Model and Regression-Genomic Control) [38], [39]. This approach has been used in multiple GWAS in cattle [23], [40], [41]. The advantage of this approach especially in livestock is that it accounts for cryptic population structure caused by the presence of closely related animals [38]. In the absence of pedigree information, GRAMMAR-GC infers relationships through genomic marker data. Following the approach of Aluchenko et al. [38], in the first step phenotypes were corrected by accounting for familial dependence among individuals using: 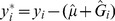
(2)where 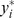 is the so-called “environmental residual” and *y_i_* is the binary phenotype of *i^th^* animal, µ is the overall mean, 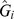 is the estimated polygenic contribution. In the second step, these familial correlation-free residuals were used as dependent quantitative traits for association analysis of each SNP using a linear regression model: 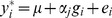
(3)where 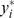 is as defined before, *g*_i_ is the genotype of the *i* ^th^ individual at the marker under study, *α_j_* is the effect of *j* ^th^ SNP and *e_i_* is the random residual for the *i* ^th^ individual. In the third step, genomic control (GC) is applied to correct the test statistic using a deflation factor (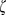) calculated by: 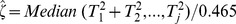
(4)where 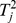 is the observed chi-squared (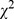) statistic for the *j^th^* SNP and 0.465 is the expected median of 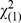 distribution with a non-central variance. 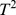 for each SNP is calculated by
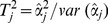
(5)where 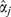 is the effect of *j^th^* SNP. 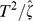 is compared with 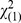 to determine whether the locus is significantly associated with the quantitative trait. The deflation factor is estimated in the same way as inflation factor 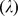 in conventional GC method [42] with the difference that 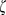
<1 in contrast to 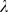 that is constrained to be > 1. This difference is due to the regression of residuals instead of original trait on n loci in step 2. *Polygenic* [43] and *qtscore* [38], [39] functions of the R GenABEL package were used for association analysis. Two P-value thresholds of 5×10^−7^ and 5×10^−5^ were considered for genome-wide “strong” and “moderate” associations [44]. The Manhattan plot of minus log_10_ (P-value) against chromosomes was drawn using an in-house script in R [45]. The quantile-quantile (Q-Q) plot of observed P-values against expected P-values was generated to evaluate the overall genome-wide significance.

To validate the associations suggested by discovery GWAS, validation data (31,065 SNPs and 180 animals) were analyzed by single-marker GWAS procedures described above. For validation, strong and moderate associations suggested by discovery GWAS were required to meet two criteria in the validation analysis: P<0.01 and same direction for estimated effects.

**Bayesian GWAS.** In contrast to traditional single-marker regression based GWAS that fits one marker at a time, Bayesian methods simultaneously fit many markers and take into account the linkage disequilibrium (LD) relationships between markers. Bayesian methods were originally adopted for genomic prediction of breeding values [46], however, in recent years they have been applied for GWAS as well e.g. [47], [48]. We used the Bayes C threshold model implemented in GenSel [49] for both mapping quantitative trait loci (QTL) of *MAP* infection as an alternative approach to single-marker GWAS and developing a multi-marker model to predict new phenotypes (risk assessment). Bayesian methodology combines prior information of marker effects with information from data to draw inferences from posterior distributions using Markov Chain Monte-Carlo (MCMC) sampling [50]. In Bayes C, a common variance is assumed for all SNPs and its advantage over other Bayesian approaches is that it is less sensitive to priors of genetic and residual variances [50]. The threshold model in Bayes C with a probit link function for categorical binary traits described [51] as:
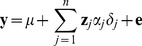
(6)where **y** is the liability vector for case/control observations, µ is the overall mean, *n* is number of SNPs, ***z*** represents a column vector of genotype covariates at SNP*_j_* (AA = –10, AB = 0 and BB = 10), 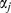
is the allele substitution effect of SNP*_j_*, 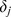 is a an indicator variable for presence (1) or absence (0) of *j^th^* SNP in the model and ***e*** is the vector of random residual effect assumed normally distributed ∼*N* (0, **I**) **I** being an identity matrix. The proportion of SNPs with no effect (parameter π) was assumed to be 0.999. Therefore, 33 SNPs (0.1% of 32,587 SNPs) were assumed to contribute to genetic variance in any MCMC iteration. A high value of π was chosen to allow only regions with strongest associations to be identified. In Bayes C when a SNP is present in the model (i.e. 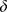 = 1), 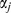
is assumed to be normally distributed ∼*N* (0, 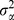) conditional on 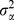, whereas when the SNP is not present (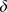 = 0) 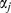 is zero. Residual variance was set to 1. Assuming an average heritability of 0.1 for susceptibility to *MAP*-infection in dairy cattle [10], [11], [12], [13], [14], [15], 0.11 was used as a prior for genetic variance (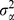). 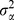 was assumed to have a scaled inverse chi-squared distribution with 4 degrees of freedom and scale parameter 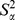. A total of 41,000 MCMC cycles with 1,000 burn-in cycles were implemented.

Because of LD between markers in the vicinity of a QTL, the effect of a QTL may be distributed over nearby markers. Consecutive 1 Mb non-overlapping windows (genome windows) along the bovine genome were used to calculate the cumulative effects of markers within windows [52]. SNPs were allocated to consecutive genome windows using physical map order derived from Bovine genome assembly UMD 3.1. The window effect in GenSel is expressed as the percentage of total genetic variance contributed by each window. Percentages of explained genetic variance by windows were plotted against chromosomes using R [45]. Model frequency of a marker defined as the proportion of fitted models which included that marker was used to infer associations. SNPs with the highest model frequency in top windows are potentially associated with the phenotype under study. In Bayesian GWAS, limiting the proportion of false positives (PFP) among all positive values is an approach that can be used to correct for multiple testing [53]. Therefore, if SNPs with model frequency ≥ 0.90 were deemed significant, PFP would be ≤ 0.10.

**Genomic prediction.** A multi-SNP model was developed by Bayes C analysis in the discovery data (training set). Using the *predict* function in GenSel the genomic estimated breeding values (GEBV) were obtained for 180 animals in the validation data (testing set). The efficacy of predicted GEBVs in correctly ranking cases and controls was evaluated by Receiver operator characteristic (ROC) analysis implemented in ROCR package of R [54]. The ROC curve plots the true-positive rate (sensitivity) against the false-positive rate (1- specificity) which graphically depicts the accuracy with which a risk classification score (GEBV, in this study) predicts the binary outcome (infection status) across a full range of thresholds. The area under ROC curve (AUC) is a statistic that quantifies the classification power of the SNP model, where values of 1.0 and 0.5 reflect perfect classification and random assignment [55].

As an alternative, a 10-fold cross-validation was also performed using the discovery data. 450 cases and 439 controls were randomly divided into 10 approximately equal subgroups. Nine subgroups were assigned to a training set while the remaining subgroup was considered as a testing set. For each replication, we used the training set to construct a SNP model which subsequently was tested in the testing set. This procedure was repeated 9 additional times with a unique testing set each time. Ten different models were constructed in GenSel using Bayes C (same input parameters used in initial Bayesian GWAS). The GEBVs of animals in the testing sets were calculated using GenSel *predict* and the efficacies of SNP models were evaluated by comparing AUCs.

**Combined analyses.** The discovery (N = 889) and validation (N = 180) data were merged to enhance the power of analyses. The combined data comprised of 1,069 animals and 32,375 SNPs. A total of 212 SNPs were excluded in QC: 175 SNPs due to MAF <0.01, 33 SNPs for call rate <0.95 and 4 SNPs were out of HWE (P<10^−6^). Population stratification, single-marker GWAS, Bayesian GWAS and 10-fold cross-validation were performed in the manner described before.

## Results

### Single-marker GWAS (discovery data)

**Figure 1:**
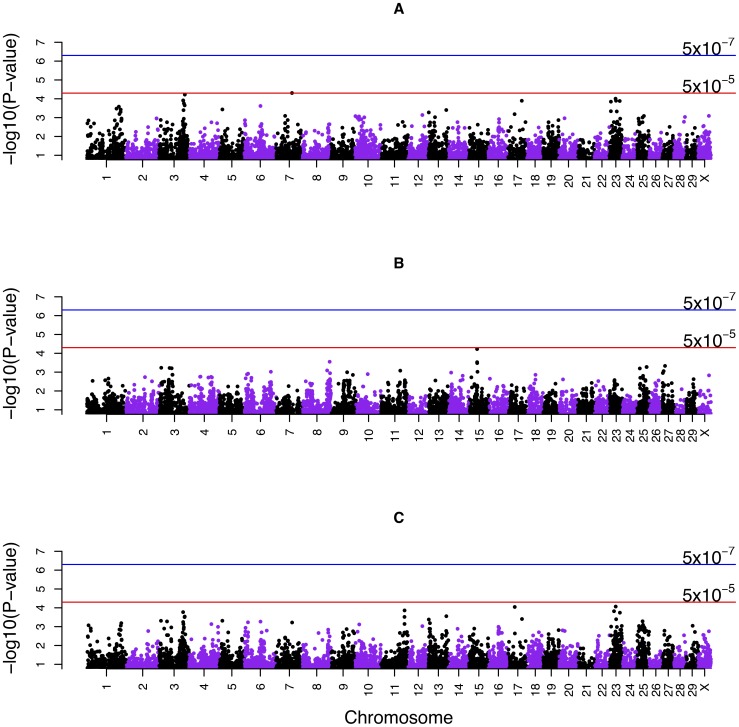
Manhattan plots displaying the results of single-marker genome-wide association analysis (GRAMMAR-GC) for susceptibility to *MAP* infection. A) Discovery data (32,587 SNPs) B) Validation data (31,065 SNPs) C) Combined data (32,375 SNPs). Y-axis represents –log_10_ of P-values corrected by genomic control (GC) and X-axis represents chromosomes. Thresholds represent P-values of 10^−4^ and 5×10^−4^ for moderate and suggestive associations, respectively.

Appearance of a single cluster in the MDS plot suggested the absence of population substructure in the discovery data (Figure S1-A). The deflation factor (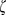) was estimated to be 0.96 (SE = 9×10^−5^). The GC-corrected P-values for the majority of SNPs corresponded well to the expected P-values under H_0_ of no association, with a few departures indicating association with the trait under study (Figure S2-A). No SNP passed the threshold of strong association (Figure 1-A). The most significant SNP was identified on BTA7 position 68 Mb (P = 4.9×10^−5^) surpassing the threshold for moderate association (Table 2). The second most significant SNP (P = 5.9×10^−5^) was located on BTA3 (107 Mb) and failed to pass the moderate threshold (Figure 1-A, Table 2). The 20 most significant SNPs (P<5×10^−4^) were located on 8 chromosomes including BTA7, 3, 23, 17, 6, 1, 5 and 13 (in order of significance) (Table 2). On BTA3 a total of six SNPs covering 101 to 107 Mb were identified (Table 2). These six SNPs represented four distinct genomic regions based on LD between pairs of SNPs; three SNPs (at 100.9 Mb, 101 Mb and 102.2 Mb) were in high LD (average pair wise r^2^ = 0.67) representing one genomic region and the other three SNPs each represented one region. Also, six SNPs were identified on BTA23 representing a total of four regions including 27.7 Mb, 29.3–32.6 Mb (r^2^ = 0.63), 44.4 Mb and one region at 7.8 Mb (r^2^ = 0.54 for SNPs at 7.84 and 7.87 Mb) (Table 2). BTA1 contained three SNPs located at positions 125.6, 135.3 and 141.9 Mb (P<5×10^−4^) representing three genomic regions based on a relatively low average pair wise r^2^ (0.25) (Table 2).

**Table 2:** Results of single-marker (GenABEL) and Bayesian (GenSel) genome-wide association analysis for susceptibility to *MAP* infection in Jersey cattle (discovery data, N = 889)^1^.

SNP	BTA^5^	Position^6^	A1^7^	A2^8^	Effect B (SE)^9^	P-value^10^	Q.2 (Cases)^11^	Q.2 (Controls)^12^	GenSel Rank^13^	Window (SNPs)^14^	%GV^15^	P> avg.^16^	Model freq.^17^
**Common SNPs** 2													
BTA-109542-no-rs	7	68 461 305	G	A	–0.128 (0.032)	4.9×10^−5^	0.09	0.16	11	854 (8)	0.97	0.12	0.12
Hapmap53765-ss46526662	3	107 097 209	G	A	–0.086 (0.022)	5.9×10^−5^	0.33	0.43	2	406 (13)	3.19	0.21	0.19
ARS-BFGL-NGS-11887	23	27 776 075	A	G	–0.083 (0.022)	9.8×10^−5^	0.36	0.45	1	2199 (19)	3.32	0.26	0.21
ARS-BFGL-NGS-109837	3	100 901 542	A	C	–0.082 (0.022)	1.2×10^−4^	0.42	0.51	7	399 (19)	1.49	0.15	0.12
Hapmap51790-BTA-103080	3	101 076 596	A	G	–0.083 (0.022)	1.2×10^−4^	0.39	0.49	4	400 (13)	1.99	0.17	0.11
ARS-BFGL-BAC-35219	23	29 316 835	G	A	–0.103 (0.027)	1.3×10^−4^	0.16	0.23	8	2201 (9)	1.38	0.14	0.13
ARS-BFGL-NGS-100555	17	57 131 089	A	G	0.083 (0.022)	1.3×10^−4^	0.42	0.32	3	1818 (16)	2.77	0.24	0.21
BTA-56690-no-rs	23	44 458 259	A	G	–0.126 (0.034)	1.3×10^−4^	0.09	0.15	20	2216 (18)	0.56	0.08	0.04
ARS-BFGL-NGS-115177	23	7 874 236	A	G	–0.080 (0.022)	1.4×10^−4^	0.37	0.47	6	2179 (14)	1.87	0.17	0.11
ARS-BFGL-NGS-111440	3	104 048 383	A	G	–0.082 (0.022)	1.8×10^−4^	0.39	0.48	10	403 (14)	1.23	0.11	0.08
BTB-00148619	3	106 442 016	A	G	–0.078 (0.022)	2.2×10^−4^	0.36	0.45	13	405 (15)	0.87	0.09	0.07
BTA-30686-no-rs	6	60 692 672	A	C	0.079 (0.022)	2.4×10^−4^	0.42	0.33	5	727 (10)	1.91	0.17	0.15
BTA-75232-no-rs	5	10 104 621	G	A	–0.074 (0.021)	3.7×10^−4^	0.44	0.53	9	554 (14)	1.24	0.12	0.12
ARS-BFGL-NGS-114203	1	141 903 958	A	G	–0.093 (0.027)	3.7×10^−4^	0.15	0.23	17	141 (13)	0.59	0.10	0.05
**SNPs (GenABEL only)** 3													
ARS-BFGL-NGS-78666	1	135 366 450	A	G	–0.125 (0.035)	2.6×10^−4^	0.08	0.13	-	-	-	-	-
Hapmap50536-BTA-99808	1	125 641 702	G	A	–0.118 (0.033)	3.3×10^−4^	0.08	0.14	-	-	-	-	-
ARS-BFGL-NGS-38764	13	77 546 073	G	A	–0.186 (0.053)	3.9×10^−4^	0.03	0.06	-	-	-	-	-
ARS-BFGL-NGS-2973	3	102 289 610	C	G	–0.076 (0.022)	4.0×10^−4^	0.43	0.52	-	-	-	-	-
ARS-BFGL-NGS-109956	23	7 841 643	G	A	–0.081 (0.023)	4.6×10^−4^	0.25	0.32	-	-	-	-	-
ARS-BFGL-NGS-19381	23	32 621 505	A	C	–0.092 (0.027)	4.7×10^−4^	0.16	0.23	-	-	-	-	-
**SNPs (GenSel only)** 4													
BTB-00643802	16	48 725 717	-	-	-	-	-	-	12	1727 (18)	0.89	0.12	0.03
BTB-01486995	6	1 305 535	-	-	-	-	-	-	14	668 (18)	0.81	0.11	0.03
Hapmap40033-BTA-93880	19	61 125 348	-	-	-	-	-	-	15	1963 (15)	0.74	0.10	0.03
ARS-BFGL-NGS-38096	X	136 472 106	-	-	-	-	-	-	16	2642 (10)	0.74	0.09	0.06
Hapmap55445-rs29014484	10	1 979 167	-	-	-	-	-	-	18	1122 (12)	0.58	0.08	0.05
Hapmap25546-BTA-137359	23	35 309 434	-	-	-	-	-	-	19	2207 (15)	0.56	0.05	0.03

1 Only the twenty most significant SNPs are shown.

2 SNPs identified commonly by both GenABEL and GenSel analyses; order is based on P-values from GenABEL.

3 SNPs identified only by GenABEL.

4 SNPs identified only by GenSel.

5 *Bos Taurus* chromosomes.

6 Position of SNP based on Bovine genome build UMD 3.1 (in base pair).

7 Major allele.

8 Minor allele.

9 Estimated effect of allele B (fitted allele) and the standard error of the estimated effect in the parenthesis.

10 P-value corrected by genomic control approach (GC).

11 Frequency of B allele in cases.

12 Frequency of B allele in controls.

13 Rank based on percentage of genetic variance among the twenty most significant windows by GenSel analysis.

14 Number of 1-Mb non-overlapping genome window and number of SNPs within each window in the parenthesis.

15 Percentage of total genetic variance explained by 1-Mb windows.

16 Proportion of models in which the corresponding window accounted for > 0.04% of genetic variance (expected variance if each window had the same effect: 1/total number of windows  = 2,657).

17 Proportion of MCMC iterations that included the corresponding SNP.

### Single-marker GWAS (validation data)

The MDS plot for validation data (after imputation) did not show any outliers and confirmed genetic homogeneity in the population (Figure S1-B). The estimated deflation factor from GRAMMAR was 0.98 (SE = 2×10^−4^). The Q-Q plot of corrected P-values is shown in Figure S2-B. None of the 20 most significant SNPs identified in the discovery analysis were significant (P<0.01) in validation analysis (Figure 1-B). The effect directions of these SNPs were compared with validation results, and 65% of the effects were in the same direction.

### Bayesian GWAS (discovery data)

From Bayes C analysis, the mean posterior estimates of contribution of SNPs to genetic variance was 0.186 with the 95% highest posterior density (HPD) of 0.048–0.370. The genetic variance was computed from allele frequency and posterior mean of substitution effect for each SNP in 40,000 MCMC iterations. The mean posterior estimate of heritability (h^2^) of susceptibility to *MAP* infection based on SNPs was 0.153 and 95% HPD of 0.046–0.270 (Table 3).

**Table 3:** Posterior means of variance components in Bayesian genome-wide association study of susceptibility to *MAP* infection in US Jersey cattle.

Data	No. of animals	No. of SNPs	Posterior mean of genetic variance (HPD)^2^	Categorical fixed residual variance	Total variance	Posterior mean of proportion of variance explained by all SNPs (HPD)^2^
Discovery	889	32,587	0.186 (0.048–0.370)	1.00	1.18	0.153 (0.046–0.270)
Combined^1^	1,069	32,375	0.147 (0.042–0.287)	1.00	1.14	0.126 (0.040–0.223)

1 Discovery and validation data.

2 95% highest posterior density of the estimates.

**Figure 2:**
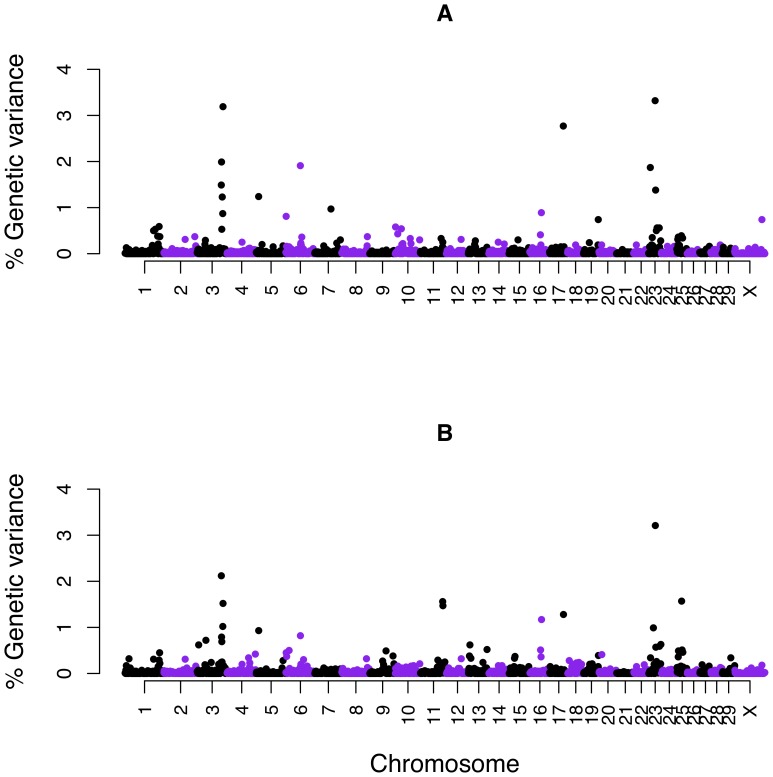
Manhattan plots displaying the results of Bayesian genome-wide association analysis (Bayes C) for susceptibility to *MAP* infection. A) Discovery data (2,657 windows) B) Combined data (2,656 windows). Y-axis represents the proportion of genetic variance explained by 1-Mb windows across the Bovine genome and X-axis represents the chromosomal location of windows.

A total of 2,657 genome windows existed along the Bovine genome with an average of 12 SNPs per window. The genome windows were sorted based on the proportion of genetic variance captured by each. In the top 20 windows, the proportion of genetic variance explained by a window ranged between 0.56% to 3.3% (Table 2). Assuming an infinitesimal model, the expected proportion of genetic variance explained by each window was ∼0.04% ([1/2,657] * 100). In total, 584 windows (22%) explained more than 0.04% of the genetic variance. Window 2199 on BTA23 (27 to 27.9 Mb) containing 19 SNPs accounted for the highest percentage of the genetic variance (3.32%) (Figure 2-A, Table 2). The probability that this window explained more than the average genetic variance was 0.26. In window 2199, ARS-BFGL-NGS-11887 at position 27.7 Mb among other SNPs, showed the highest model frequency, i. e., was included in the model in 21% of MCMC iterations (Table 2).

The top 20 windows were located on BTA23 (5 windows), BTA3 (5 windows), BTA6 (2 windows) and BTA17, 5, 7, 16, 19, X, 1 and BTA10 (one window each). To compare these results with the results of single-marker GWAS, for each window the SNP with the highest model frequency (the most influential SNP) was chosen to represent the window. For BTA3 and BTA23 the results of Bayesian GWAS corresponded with the results of single-marker GWAS (Table 2). The loci that were among the 20 most significant in the Bayesian analysis (GenSel) but not the GenABEL analysis included SNPs on BTA16 (48 Mb), 6 (1 Mb), 19 (61 Mb), × (136 Mb), 10 (1 Mb) and 23 (35 Mb)(Table 2). The most significant SNP based on GenABEL analysis, BTA-109542-no-rs on BTA7, ranked 11^th^ in the GenSel analysis. Considering the 20 most significant SNPs in each, the results of the two analyses (GenABEL and GenSel) were in high agreement (70% of loci in common). In total, 10 SNPs had model frequency > 0.10. Assuming these SNPs to be positive results, we would expect at least one of these SNPs to be truly associated with *MAP* infection (PFP <0.90).

### Genomic prediction (validation data)

**Figure 3:**
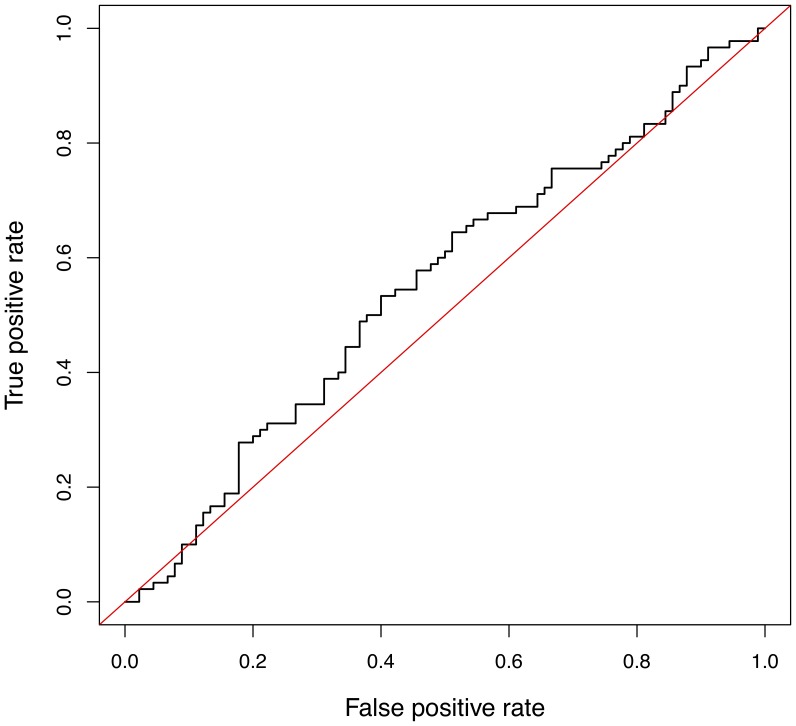
Receiver Operating Characteristic (ROC) curve for validation data. Multi-SNP model was developed by Bayes C analysis of discovery data and tested in classifying 180 case vs. control animals in validation data. Broken line represents the model. Area under ROC curve is equivalent to the probability of correctly assigning a random pair of observations (positive and negative) to case and control. The diagonal represents a model with no predictive ability (AUC = 0.5).

**Figure 4:**
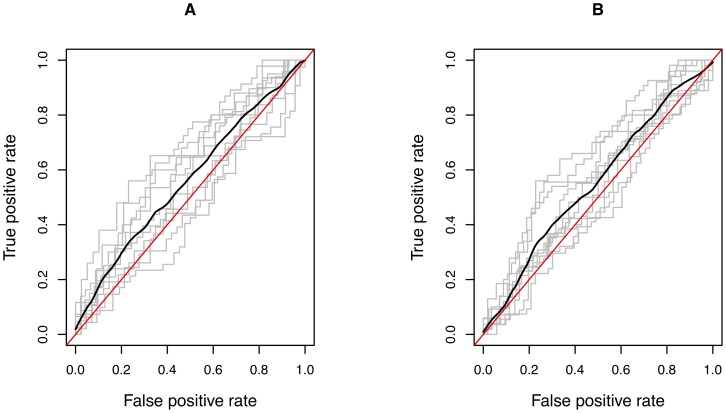
Receiver Operating Characteristic (ROC) curve for 10 fold cross-validation. A) Discovery data (32,587 SNPs) B) Combined data (32,375 SNPs). Ten sets of training and testing subsets were created. Multi-SNP models were developed by Bayes C analysis in training sets and were validated in testing set. Each broken line represents one model and solid bold line is the average area under curve (AUC) of all models. AUC is equivalent to the probability of correctly assigning a random pair of observations (positive and negative) to case and control. The diagonal represents a model with no predictive ability (AUC  = 0.5).

The marker effect estimates from the Bayesian analysis were used to predict the genomic merit of 180 animals in the validation set. The predicted genomic merit was used to rank paired case and control samples which was compared with observed phenotype by ROC analysis. The predictive ability of the model was low. The AUC of the SNP model was 0.55 (Figure 3). The AUC from 10-fold-cross validation using discovery data was similar, ranging between 0.47 to 0.67 (average 0.56) (Figure 4-A).

### Combined analyses

**Single-marker GWAS.** No population substructure existed in the combined data set (Figure S1-C). The deflation factor from GRAMMAR-GC was 0.94 (SE = 1.6×10^−4^). The Q-Q plot of corrected P-values is shown in Figure S2-C. No SNP passed the strong or moderate thresholds of association (Figure 1-C, Table 4). The two most significant SNPs were located on BTA23 at 27.7 Mb (third most significant in discovery analysis by GenABEL) and BTA17 position 26.3 Mb (not among the 20 most significant results in discovery data) (P<10^−4^) (Figure 1-C, Table 4).

**Table 4:** Results of single-marker (GenABEL) and Bayesian (GenSel) genome-wide association analysis for susceptibility to *MAP* infection in Jersey cattle (combined data, N = 1,069)^1.^

SNP	BTA^5^	Position^6^	A1^7^	A2^8^	Effect B (SE)^9^	P-value^10^	Q.2 (Cases)^11^	Q.2 (Controls)^12^	GenSel Rank^13^	Window (SNPs)^14^	%GV^15^	P> avg.^16^	Model freq.^17^
**Common SNPs** 2													
ARS-BFGL-NGS-11887	23	27 776 075	A	G	–0.077 (0.02)	8.5×10^−5^	0.35	0.44	1	2199 (18)	3.21	0.29	0.23
Hapmap45793-BTA-114539	11	93 027 457	A	T	0.081 (0.022)	1.3×10^−4^	0.32	0.25	6	1318 (8)	1.47	0.15	0.16
ARS-BFGL-NGS-92339	23	19 397 902	C	A	–0.10 (0.027)	1.5×10^−4^	0.12	0.18	10	2191 (18)	0.99	0.12	0.09
ARS-BFGL-NGS-109837	3	100 901 542	A	C	–0.072 (0.020)	1.6×10^−4^	0.43	0.51	2	399 (18)	2.12	0.19	0.15
BTA-56690-no-rs	23	44 458 259	A	G	–0.109 (0.030)	1.8×10^−4^	0.10	0.15	19	2216 (18)	0.59	0.08	0.04
Hapmap53765-ss46526662	3	107 097 209	G	A	–0.07 (0.020)	2.9×10^−4^	0.33	0.41	5	406 (13)	1.52	0.12	0.08
ARS-BFGL-NGS-20015	11	92 692 190	G	A	0.071 (0.020)	3×10^−4^	0.43	0.35	4	1317 (15)	1.56	0.16	0.08
BTB-00148619	3	106 442 016	A	G	–0.069 (0.020)	3.1×10^−4^	0.36	0.45	9	405 (15)	1.02	0.11	0.09
ARS-BFGL-NGS-100555	17	57 131 089	A	G	0.07 (0.020)	3.9×10^−4^	0.42	0.33	7	1818 (16)	1.28	0.13	0.1
ARS-BFGL-BAC-35219	23	29 316 835	G	A	–0.085 (0.025)	4.0×10^−4^	0.17	0.23	20	2201 (9)	0.57	0.07	0.04
BTA-75232-no-rs	5	10 104 621	G	A	–0.066 (0.010)	4.8×10^−4^	0.45	0.54	11	554 (14)	0.93	0.1	0.1
ARS-BFGL-NGS-26988	3	6 183 874	G	A	–0.081 (0.024)	4.8×10^−4^	0.18	0.24	18	305 (11)	0.62	0.07	0.06
ARS-BFGL-NGS-95270	25	19 724 505	G	A	–0.073 (0.022)	5.1×10^−4^	0.25	0.52	3	2308 (13)	1.57	0.16	0.08
BTA-30686-no-rs	6	60 692 672	A	C	0.068 (0.020)	5.4×10^−4^	0.42	0.34	12	727 (10)	0.82	0.1	0.09
ARS-BFGL-NGS-117495	3	36 531 738	C	A	0.069 (0.020)	5.2×10^−4^	0.40	0.32	14	335 (5)	0.72	0.08	0.08
Hapmap51790-BTA-103080	3	101 076 596	A	G	–0.067 (0.020)	5.6×10^−4^	0.40	0.48	13	400 (12)	0.79	0.09	0.05
**SNPs (GenABEL only)** 3													
ARS-BFGL-BAC-18843	17	26 318 464	A	G	–0.194 (0.051)	9.0×10^−5^	0.02	0.06	-	-	-	-	-
ARS-BFGL-NGS-38764	13	77 546 073	G	A	–0.183 (0.052)	2.7×10^−4^	0.02	0.05	-	-	-	-	-
BTB-01324240	13	1 867 669	A	G	–0.161 (0.047)	4.1×10^−4^	0.03	0.06	-	-	-	-	-
BTA-22128-no-rs	6	13 040 713	A	G	–0.116 (0.035)	5.8×10^−4^	0.06	0.11	-	-	-	-	-
**SNPs (GenSel only)** 4													
BTB-00643802	16	48 725 717	-	-	-	-	-	-	8	1727 (17)	1.17	0.11	0.03
ARS-BFGL-NGS-2973	3	102 289 610	-	-	-	-	-	-	15	401 (12)	0.69	0.07	0.04
ARS-BFGL-NGS-31524	23	50 747 363	-	-	-	-	-	-	16	2222 (17)	0.63	0.08	0.03
BTB-01124458	13	6 683 656	-	-	-	-	-	-	17	1430 (11)	0.62	0.07	0.04

1 Only the twenty most significant SNPs are shown.

2 SNPs identified commonly by both GenABEL and GenSel analyses; order is based on P-values from GenABEL.

3 SNPs identified only by GenABEL.

4 SNPs identified only by GenSel.

5 *Bos Taurus* chromosomes.

6 Position of SNP based on Bovine genome build UMD 3.1 (in base pair).

7 Major allele.

8 Minor allele.

9 Estimated effect of allele B (fitted allele) and the standard error of the estimated effect in the parenthesis.

10 P-value corrected by genomic control approach (GC).

11 Frequency of B allele in cases.

12 Frequency of B allele in controls.

13 Rank based on percentage of genetic variance among the twenty most significant windows by GenSel analysis.

14 Number of 1-Mb non-overlapping genome window and number of SNPs within each window in the parenthesis.

15 Percentage of total genetic variance explained by 1-Mb windows.

16 Proportion of models in which the corresponding window accounted for > 0.04% of genetic variance (expected variance if each window had the same effect: 1/total number of windows  = 2,657).

17 Proportion of MCMC iterations that included the corresponding SNP.

The 20 most significant SNPs by combined analysis (P<6×10^−4^), representing 8 genomic regions, were mapped to 8 chromosomes including BTA23 (4 SNPs), 11 (2), 3 (6), 17 (2), 5, 25, 6 (2) and 13 (2) (ordered by P-value) (Table 4). Comparing single-marker GWAS results of the combined and discovery data, the most significant SNP on BTA7 (P = 4.9×10^−5^) in discovery analysis declined in significance in the combined analysis (Table 2 & 4). Likewise, the SNPs located on BTA1 and at 7 Mb of BTA23, also declined in significance (Table 2 & 4). A total of 11 SNPs including 4 SNPs on BTA3, 4 SNPs on BTA23, one SNP on each BTA5, 6, 13 and 17 25 were identified by both analyses (Table 2 & 4).

**Bayesian GWAS.** A total of 2,656 genome windows containing 32,375 SNPs were used to estimate the genetic variance explained by SNPs in combined data. The mean posterior estimate of genetic variance was 0.147 (95% HPD: 0.042–0.287). The mean posterior estimate of h^2^ was 0.126 with 95% HPD interval from 0.040 to 0.223 (Table 3).

The 20 most significant windows were located on 9 chromosomes including BTA23 (5 windows), 3 (7), 25, 11 (2), 17, 16, 5, 6, and 13 (Table 4). SNPs on BTA23 were located at 19.3, 27.7, 29.3, 44.4 and 50.7 Mb. Five out of seven windows on BTA3 were contiguous: three windows 399, 400, and 401 (respectively, these included SNPs at 100, 101 and 102 Mb); and two windows 405 and 406 (respectively, these included SNPs at 106 and 107 Mb). The highest percentage of genetic variance (3.2%) was explained by window 2199 on BTA23 covering 27 to 28 Mb (Figure 2-B, Table 4). In total, 603 out of 2656 windows (23%) explained ≥ 0.04% of genetic variance (expected genetic variance under the infinitesimal model). Similar to the results of Bayesian GWAS for discovery data, SNP ARS-BFGL-NGS-11887 in window 2,199 had the highest model frequency (0.23), slightly higher compared to model frequency of 0.21 in the discovery results (Table 2 & 4).

Comparing the results of Bayesian analysis between combined and discovery data, 11 SNPs [BTA23 (3 SNPs), BTA3 (4) and BTA17, 6, 5 and 16] were common between the 20 most significant SNPs in the two analyses (Table 2 & 4). New loci that appeared within the 20 most significant windows by combined analysis included loci on BTA23 (19 and 50 Mb), BTA3 (6, 36 and 102 Mb), BTA11 (92 and 93 Mb), BTA13 (6 Mb) and BTA25 (19 Mb) (Table 4). Model frequencies of SNPs in most cases were equal or smaller in combined analysis compared to discovery. Likewise, the percentage of explained genetic variance by each window was generally smaller in combined analysis (Table 2 & 4). Comparing the results of Bayesian GWAS with single-marker GWAS for combined data, 16 out of 20 most significant SNPs were common between both analyses (80% concordance) (Table 4).

### Genomic prediction (cross-validation)

In a ten-fold cross-validation, the predictive abilities of models developed by training with 90% of combined data were evaluated in the remaining 10%. AUC ranged from 0.46 to 0.65 for the models (ROC curves in Figure 4-B). Average AUC across 10 models was 0.55.

## Discussion

This is the first genome-wide association study for susceptibility to paratuberculosis in Jersey cattle. Previously, GWAS [21], [22], [23], [24], [26] and a meta-analysis [41] were conducted to identify loci responsible for susceptibility to this condition in Holsteins. These studies have found evidence for association on multiple and varying chromosomal locations.

The proportion of variance explained by all SNPs across the genome (0.15 and 0.12) was in the range of pedigree-based heritability estimates (0.08 to 0.27, unpublished data). This suggests that markers captured most of the additive genetic variation through LD with QTL. However, the previous pedigree-based heritability estimates are from studies of Holstein cattle. No report is available for the heritability of susceptibility to paratuberculosis in Jersey cattle. Studies have shown that susceptibility to *MAP* infection may differ between breeds [56], [57]. Sorge et al., (2011) reported that Jersey cows are 1.4 times more likely to test positive to milk ELISA than Holstein cows [56]; however, this conclusion is questionable as breeds were confounded with herds in their data. To capture loci with the largest genetic contribution a high value of the π parameter (0.999) was used in the current analysis; with a smaller π, the proportion of genetic variance explained by all SNPs might be increased.

The significance level in single-marker and Bayesian GWAS was generally low; no SNPs surpassed the threshold of strong association in separate or combined analyses. Likewise, the highest model frequency of SNPs in most significant windows for both discovery and combined analyses was 0.23. The low model frequencies resulted in higher PFP. For π = 0.999, a randomly chosen locus would *a prior*i be expected to have non-zero effect in 0.1% of MCMC samples. A model frequency of 0.23 indicates that particular SNP had a non-zero effect in 23% of MCMC samples. The relatively low significance of identified SNPs can be explained by the complex genetic architecture of susceptibility to paratuberculosis and that there are many genes with small effects influencing this disease. The power of GWAS to identify SNPs with small effect size is limited, however, this limitation may be overcome in studies of larger scale.

The results of single-maker GWAS on discovery data showed evidence for association on BTA23 and BTA3 as multiple SNPs on these chromosomes in relatively close proximity were among the 20 most significant SNPs. There was a relatively high correspondence between the results of single-marker and Bayesian GWAS; 70% and 80% of the 20 most significant SNPs by GenABEL were also among 20 most significant SNPs by GenSel for discovery and combined analyses, respectively. The first three windows from Bayesian analysis of discovery data explained ∼10% of genetic variance while with combined data ∼7% of genetic variance was explained by the top three windows. From both GenABEL and GenSel analyses, combining data enhanced the significance of only a few SNPs.

There is some correspondence between the results of this study and previous GWAS in Holsteins. The closest correspondence is for SNP ARS-BFGL-NGS-19381 (BTA23, 32.6 Mb). The nearest SNPs to this position that were identified in Holsteins were located at 32.1 and 32.2 Mb on BTA23 [22], [41]. The 32 Mb region of BTA23 may be a case of a genomic region commonly associated with *MAP* infection across two breeds. Genes within 1 Mb of this location are four members of solute carrier family 17 (SLC17 A1, A2, A3 and A4) located between 31.7– 31.8 Mb. The 1 Mb distance was chosen based on the extent of linkage disequilibrium in cattle. Kim and Kirkpatrick (2009) showed that for pairs of markers with relatively high LD (r^2^ = 0.4–0.6) the median physical distance was ∼1.1 Mb. However, for Jersey with smaller population size and higher inbreeding the extent of LD may be even higher. Association of SNPs in SLC11A1 (another member of SLC family) with *MAP* infection has been reported [58]. SLC11A1 and SLC17A1 both encode membrane transport proteins and mutations in these genes have been associated with inflammatory diseases such as Crohn’s and Gout diseases [59], [60]. SLC17A1can be considered a potential candidate gene for predisposition to *MAP* infection in cattle.

For validation of GWAS results the ideal situation is using samples from a population independent from the discovery population with the same phenotype that was used in the discovery stage. In this study, neither of these requirements was possible. Our validation data failed to replicate the results of discovery GWAS. One reason for this might be use of a different case definition; FC+ and ELISA+/FC+ may represent distinct phenotypes i.e. loci responsible for a cow’s ability for *MAP* shedding may be different from loci underlying humoral response to the pathogen. Another limitation is the small number of samples used in the validation data set.

The predictive ability of the models developed by the Bayesian approach was low. Given that an AUC of 0.50 represents random guessing, an AUC of 0.55 or 0.56 is a weak classifier. The efficacy of the multi-marker model developed by Kirkpatrick et al. (2010) for prediction of susceptibility to *MAP* infection in Holsteins was 0.73 by AUC in cross-validation analyses [22]. It has been shown that the accuracy of genetic tests for prediction of disease susceptibility is limited by heritability and disease prevalence [55], [61]. For low heritability traits even if GEBVs are 100% accurate, prediction of unobserved phenotypes from genomic data will never be accurate [62]. It would be of interest to study the effect of heritability and prevalence on the maximum accuracy that can be obtained by multi-marker models for prediction of the risk of *MAP* infection.

Combining the results of GRAMMAR-GC and Bayes C for discovery and combined data, nine SNPs distributed on four chromosomes were commonly identified by all four analyses. These SNPs include ARS-BFGL-NGS-118877 (27.7 Mb), ARS-BFGL-BAC-35219 (29.3 Mb) and BTA-56690-no-rs (44.4 Mb) on BTA23; ARS-BFGL-NGS-109837 (100.9 Mb), Hapmap51790-BTA-103080 (101.1 Mb), BTB-00148619 (106.4 Mb) and Hapmap53765-ss46526662 (107 Mb) on BTA3; ARS-BFGL-NGS-100555 (57.1 Mb) on BTA17, and BTA-30686-no-rs (60.6 Mb) on BTA6. In most cases potentially relevant candidate genes are in near proximity (i.e. within 1 Mb). ARS-BFGL-NGS-11887 is located in the region of major histocompatibility complex (MHC) class I gene clusters (27.5–28.5 Mb) on BTA23. The role of multiple MHC genes (e.g. TNF super family, HLA, HLA-A, MIC1, AIF1, LTA, etc.) in the predisposition to CD in humans has been demonstrated or suggested [19], [63], [64], [65]. ARS-BFGL-NGS-11887 is located in the intronic region of TCF19 (transcription factor 19) which is highly conserved among multiple species. TCF19 is located on chromosome 6p21.3 close to MHC region in human and its potential role in the etiology of Type 1diabetes (an autoimmune disease) has been suggested [66]. The most relevant candidate gene within 1 Mb of SNP ARS-BFGL-BAC-35219 on BTA23 is ubiquitin D (UBD), alternatively known as FAT10; FAT10 modifies an inflammatory mediator that inhibits its activity during cellular response to Leprosy [67]. HIVEP1 is proximate to BTA-56690-no-rs at 44.4 Mb on BTA23 and encodes for a protein that participates in the transcriptional regulation of inflammatory target genes by binding specific DNA sequences in their promoter and enhancer regions [68]. The most relevant candidate gene close to ARS-BFGL-NGS-109837 and Hapmap51790-BTA-103080 is CCDC17 (coiled-coil domain containing 17) located between 101.05–101.06 Mb on BTA3. CCDC88B, from the same family, was suggested as the most promising candidate gene at location 11q13.1 in humans for susceptibility to Sarcoidosis (a complex inflammatory disease) [69]. Zinc finger protein 684 (ZNF 684) is in 200 kb of BTB-00148619 located at 106.4 Mb on BTA3; the variants in ZNF 365 have been associated with CD [70]. UBE2K (ubiquitin-conjugating enzyme E2K) located 20kb upstream of BTA-30686-no-rs (60.6 Mb) on BTA6 could be a potential candidate gene for susceptibility to paratuberculosis infection. UBE2L3 on human chromosome 22 has been identified as a new potential risk gene for CD which is also involved in other immune-mediated diseases [71]. ARS-BFGL-NGS-100555 is in close proximity (170 kb) to FAM109A (family with sequence similarity 109, member A) on BTA17. FAM5C from the same family has been reported to be associated with gastric cancer in humans [72]. All these SNPs had the same effect directions in both discovery and validation data supporting their validation. BTA-75232-no-rs (10.1 Mb) on BTA5 was identified by all four analyses but had different effect direction in validation data. The genes described above seem to be promising candidates for response to paratuberculosis infection.

## Conclusions

We performed a case-control genome-wide association study for infection with *Mycobacterium avium* subsp. *paratuberculosis* in Jersey cattle. Two statistical approaches were used: single-marker regression (GRAMMAR-GC) and Bayesian methodology (Bayes C) for multi-marker regression. Nine SNPs representing four chromosomes (BTA3, 6, 17 and 23) were identified by both GRAMMAR-GC and Bayes C analyses in discovery and combined (discovery and validation) data. Multi-marker prediction models were developed and tested by both cross-validation and application to the validation data set; predictive ability of the models to correctly rank cases and controls was low (55-56%) based on the area under ROC curve. The application of these models to predict the phenotypic outcome of animals in regard to JD is limited, however, they can be used for prediction of genetic merit.

## Supporting Information

Figure S1 **Multi-dimensional scaling plots.** A) Discovery data (N = 889) B) Validation data (N = 180) and C) Combined data (N = 1,069). Each animal is represented by one point. PC1 and PC2 are the first two principal components obtained from genomic kinship matrix. Distance between points represents the genetic distance between animals.
(PDF)

Figure S2 **Quantile-quantile plots of P-values from genome-wide association analysis for susceptibility to** ***MAP*** **infection.** A) Discovery data B) Validation data C) Combined data. Y-axis represents the observed P-values and X-axis the expected P-values under null hypothesis (diagonal) of no association.
(PDF)
