# Supplementary figures and images for: Genome-Wide Association Analysis and Genomic Prediction of Mycobacterium avium Subspecies paratuberculosis Infection in US Jersey Cattle
Source: PLoS One. 2014 Feb 11;9(2):e88380. doi: 10.1371/journal.pone.0088380 (PMC3921184; doi:10.1371/journal.pone.0088380)

**A**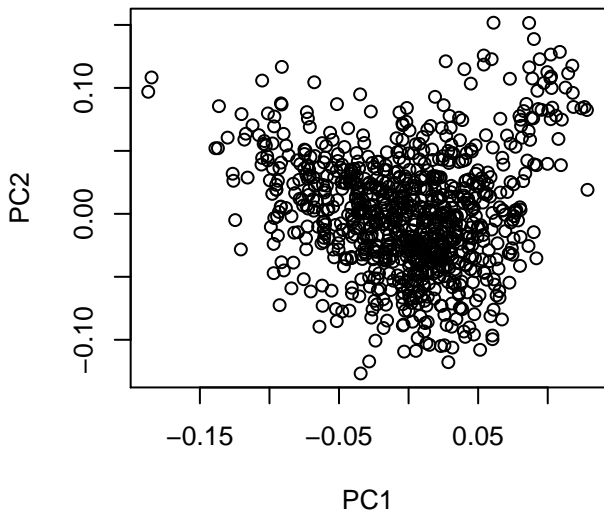**B**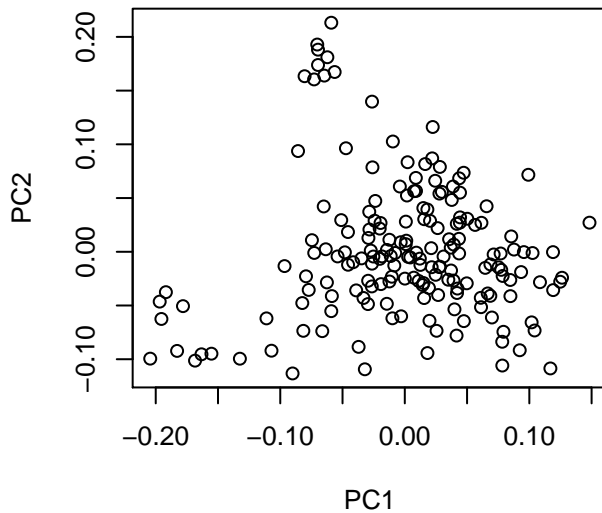**C**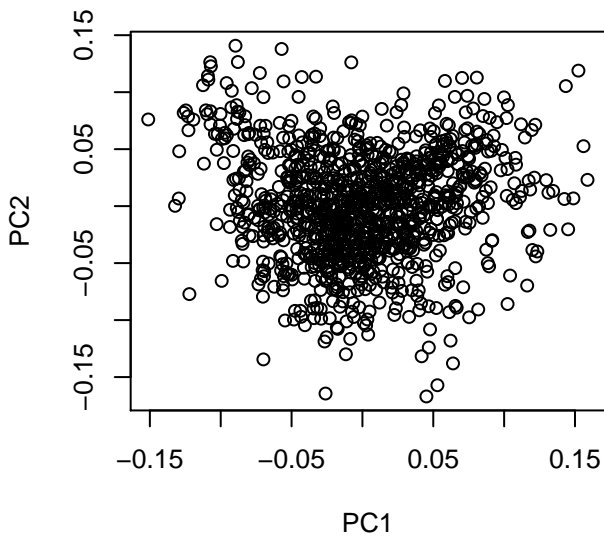

Supplement: Figure S1 — Multi-dimensional scaling plots. A) Discovery data (N = 889) B) Validation data (N = 180) and C) Combined data (N = 1,069). Each animal is represented by one point. PC1 and PC2 are the first two principal components obtained from genomic kinship matrix. Distance between points represents the genetic distance between animals. (PDF) [file pone.0088380.s001.pdf]

**A**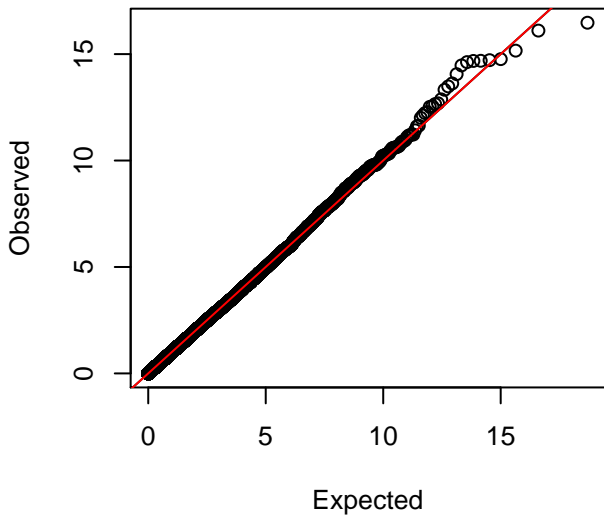**B**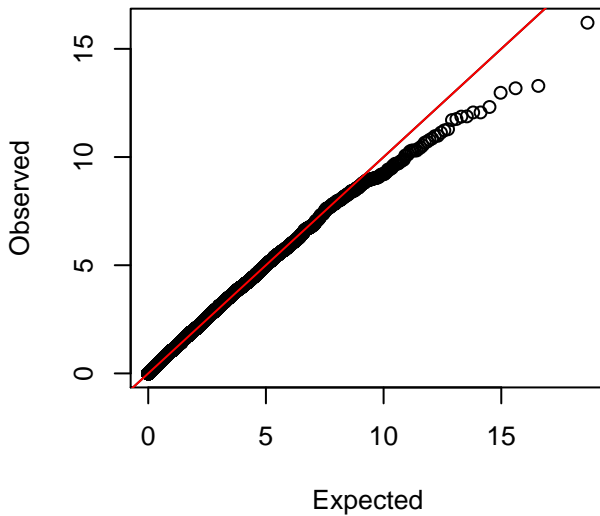**C**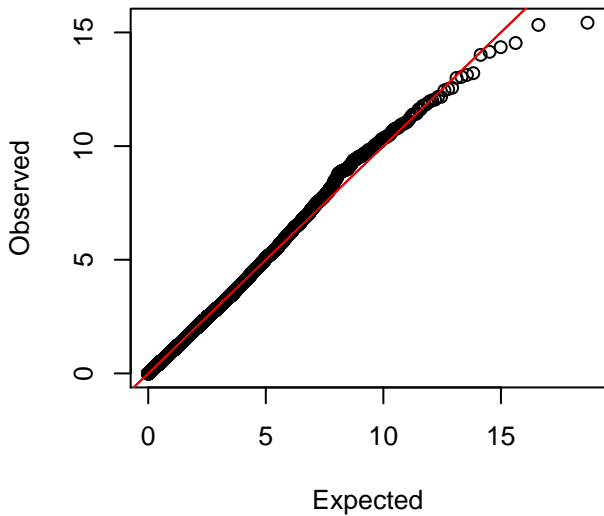

Supplement: Figure S2 — Quantile-quantile plots of P-values from genome-wide association analysis for susceptibility to MAP infection. A) Discovery data B) Validation data C) Combined data. Y-axis represents the observed P-values and X-axis the expected P-values under null hypothesis (diagonal) of no association. (PDF) [file pone.0088380.s002.pdf]
